# INVESTIGATING RELATIONSHIPS BETWEEN MUSIC REWARD AND EMPATHY ACROSS THE ADULT LIFESPAN

**DOI:** 10.1093/geroni/igad104.1405

**Published:** 2023-12-21

**Authors:** Naomi Adjei, Amy Belfi, Janelle Beadle

**Affiliations:** University of Nebraska Omaha, Omaha, Nebraska, United States; Missouri S&T, Rolla, Missouri, United States; University of Nebraska at Omaha, Omaha, Nebraska, United States

## Abstract

Music can evoke positive mood and even enhance empathy towards others. Understanding the mechanisms supporting music-based enhancements in emotion are essential for developing interventions for older adults with chronic conditions, such as Alzheimer’s disease. Previous studies have primarily focused on younger adults and have shown that trait empathy can modulate responses to music through engaging empathy-related brain networks. However, little is known about the specific relationship between music reward and trait empathy, especially across the adult lifespan. In the present study, we hypothesized that there would be a positive relationship between music reward and empathy. The sample included healthy adults 19-86 years (N=74; Mage=54.36; 62.2% female; Medu=16.2). Participants completed the Barcelona Music Reward Questionnaire (BMRQ), a 20-item questionnaire assessing music reward/appreciation and the Empathy Quotient Inventory (EQ), a 40-item questionnaire measuring multidimensional empathy (i.e., cognitive empathy, emotional reactivity, and social skills). We conducted a linear regression investigating the degree to which empathy predicted significant variance in music reward (age and gender were covariates of no interest). The overall model was significant (p=.007) and there was a significant, positive relationship between the EQ total score and the BMRQ total score (p=.004). Exploratory analyses demonstrated that the BMRQ total had positive correlations with cognitive (p=.006) and emotional empathy (p=.004). In summary, music reward was positively associated with empathy in this adult lifespan sample, adding to the literature focused on younger adulthood. Future research is needed to examine relationships between empathy and other aspects of music experience across the adult lifespan.

